# The dualistic role of Lyn tyrosine kinase in immune cell signaling: implications for systemic lupus erythematosus

**DOI:** 10.3389/fimmu.2024.1395427

**Published:** 2024-06-28

**Authors:** Elan L’Estrange-Stranieri, Timothy A. Gottschalk, Mark D. Wright, Margaret L. Hibbs

**Affiliations:** ^1^ Department of Immunology, School of Translational Medicine, Monash University, Melbourne, VIC, Australia; ^2^ Centre for Innate Immunity and Infectious Diseases, Hudson Institute of Medical Research, Clayton, VIC, Australia; ^3^ Department of Molecular and Translational Science, Monash University, Clayton, VIC, Australia

**Keywords:** SLE, lupus, Lyn tyrosine kinase, Src family kinases, immunoreceptor signaling, inhibitory signaling, autoimmune disease, autoinflammation

## Abstract

Systemic lupus erythematosus (SLE, lupus) is a debilitating, multisystem autoimmune disease that can affect any organ in the body. The disease is characterized by circulating autoantibodies that accumulate in organs and tissues, which triggers an inflammatory response that can cause permanent damage leading to significant morbidity and mortality. Lyn, a member of the Src family of non-receptor protein tyrosine kinases, is highly implicated in SLE as remarkably both mice lacking Lyn or expressing a gain-of-function mutation in Lyn develop spontaneous lupus-like disease due to altered signaling in B lymphocytes and myeloid cells, suggesting its expression or activation state plays a critical role in maintaining tolerance. The past 30 years of research has begun to elucidate the role of Lyn in a duplicitous signaling network of activating and inhibitory immunoreceptors and related targets, including interactions with the interferon regulatory factor family in the toll-like receptor pathway. Gain-of-function mutations in Lyn have now been identified in human cases and like mouse models, cause severe systemic autoinflammation. Studies of Lyn in SLE patients have presented mixed findings, which may reflect the heterogeneity of disease processes in SLE, with impairment or enhancement in Lyn function affecting subsets of SLE patients that may be a means of stratification. In this review, we present an overview of the phosphorylation and protein-binding targets of Lyn in B lymphocytes and myeloid cells, highlighting the structural domains of the protein that are involved in its function, and provide an update on studies of Lyn in SLE patients.

## 1 Systemic lupus erythematosus is a multi-system autoimmune disease

Systemic Lupus Erythematosus (SLE, lupus) is a chronic autoinflammatory disorder characterized by the production of autoantibodies, particularly towards nuclear antigen (anti-nuclear autoantibodies; ANAs) [reviewed by (1)]. ANAs can form immune complexes with self-antigen and deposit in any organ system, causing a diverse range of symptoms. SLE is known as a disease of a thousand faces and can be thought of as a prototypic autoimmune disorder, with underlying disease processes shared by many autoimmune conditions.

SLE reportedly affects one in every 1,000 to 5,000 individuals [reviewed by (2–4)]. Approximately nine times more women are impacted with the condition than men (2–4), with SLE having a higher prevalence, earlier onset, and greater severity in those with non-European ancestry (2, 4). The onset of SLE peaks in young to middle aged women (2, 3), with a later onset typically seen in men (3).

Milder forms of SLE are limited to arthritis and skin presentations, however, this can progress to life-threatening manifestations including renal disease (lupus nephritis; LN), serositis and neurological effects. Renal involvement is the most frequent serious manifestation of SLE, estimated to occur in 40-70% of SLE patients (5, 6), with one in ten LN patients progressing to end-stage renal failure within 5 years (7). Given the early onset of lupus in women, kidney disease is a significant driver of healthcare costs associated with the condition (4).

The first-line treatments for SLE include the long-term use of the antimalarial medication hydroxychloroquine to prevent future disease flares, and glucocorticoids to manage disease activity (8, 9). Other immunosuppressive agents can also be used as induction therapies or as alternatives to glucocorticoids/hydroxychloroquine when disease is refractory, including chemotherapeutics (methotrexate, cyclophosphamide) and anti-transplant-rejection medications (azathioprine, mycophenolate) (8). Achieving full remission without the need for ongoing immunosuppressive therapy is rare, and cumulative use carries significant risk of organ damage including bone disease, metabolic disorders, and retinopathy (8–11).

Advancements in immunosuppressive therapies, such as the introduction of corticosteroids and cyclophosphamide in the 1950s and 1960s, have seen an SLE patient’s prognosis greatly improve from a 10-year survival of 50-60% to 90% today (12, 13). However, there has been a lack of success in therapeutic advancement in the modern era; the 20-year overall survival of SLE patients is 75% (14, 15) and SLE is the tenth leading cause of death in young women in America, and the fifth within the African-American and Hispanic communities (16). Similarly in Australia, SLE has greater prevalence and severity amongst Asian Australians and First Nations people (17). Meta-analyses have shown that the highest increase in mortality risk is for kidney disease, followed by infection and cardiovascular disease (18, 19). An increased risk of certain cancers has also been observed in SLE (20), which is generally thought to be a result of chronic inflammation.

Research into superior therapies for SLE has been marred by a lack success (21–23). However, there have been a number of developments in recent years, including approval of two biologic therapies for SLE: belimumab to neutralize B-cell activating factor (BAFF) (24) and anifrolumab which antagonizes the type I interferon receptor (IFNARI) (25). Nonetheless, the response rate to biologics in conjunction with standard therapy is moderate, being 11% over placebo for belimumab in LN (24) and anifrolumab eliciting a 16.3% response rate in SLE patients without neuropsychiatric manifestations or active LN (25).

### 1.1 The therapeutic potential of studying Lyn in SLE

Given the modest overall response seen to biologics, there is a clear need to better understand the disease process in SLE to design more effective therapies. However, a remaining challenge in research is the diversity of SLE clinical presentations and underlying molecular causes. A key to superior therapies will therefore lie in our ability to stratify SLE patients for effective disease targeting in a precision medicine approach (26).

Lyn (Lck/yes-related novel tyrosine kinase) is a non-receptor protein tyrosine kinase that plays a unique role in immune cell signaling by propagating both activating and inhibitory pathways. Genetic manipulation studies in mice have illustrated this dichotomy, with reduction or enhancement of Lyn function giving rise to autoinflammatory diseases including lupus-like disease. Studies in SLE patients have also linked Lyn to SLE and related autoinflammatory conditions. Elucidating the mechanistic role of Lyn in SLE can therefore assist in the identification of new molecular targets for SLE therapy and shed light on whether perturbations in Lyn may serve as a biomarker for a precision medicine approach to treatment.

## 2 The dualistic role of Lyn in immune cell signaling

**Figure 1:**
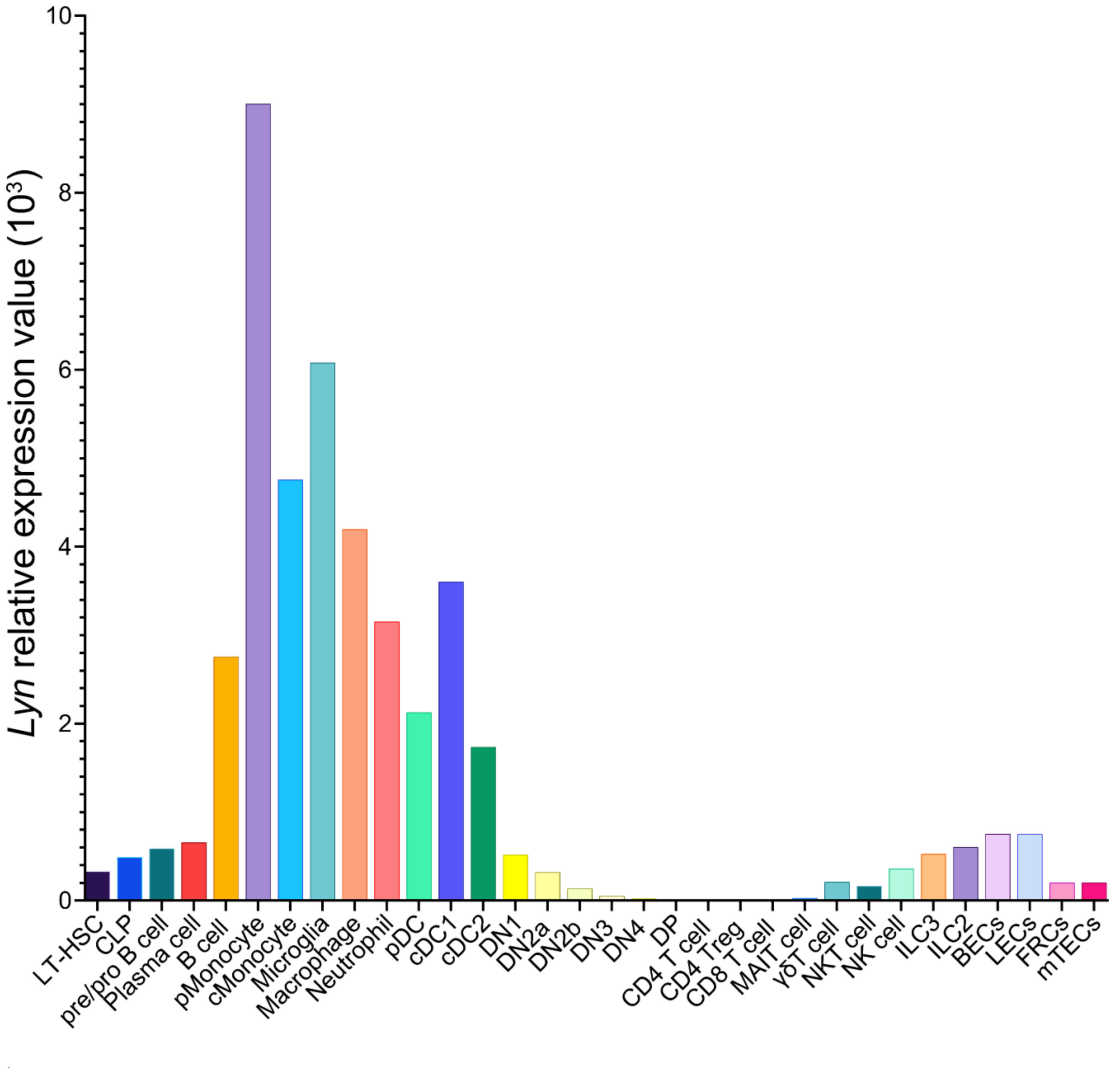
Lyn expression pattern in C57BL/6 mouse cells. Data from the Immunological Genome Project database of bulk RNA-sequencing on sorted cell populations (35). LT-HSC, long-lived hematopoietic stem cell; CLP, common lymphoid progenitor; pMonocyte, patrolling monocyte; cMonocyte, classical monocyte; pDC, plasmacytoid dendritic cell; cDC, conventional dendritic cell; DN, double negative thymocyte; DP, double positive thymocyte; MAIT, mucosal-associated invariant T cell; NK, natural killer; ILC, innate-like cell; BECs, blood endothelial cells; LECs, lymphatic endothelial cells; FRC, fibroblastic reticular cells; mTECs, medullary thymic epithelial cells.

Lyn is a member of the Src family of non-receptor tyrosine kinases (SFKs) (27). SFKs are membrane anchored enzymes that phosphorylate receptors lacking intrinsic kinase activity to initiate signal transduction. Sequence homology has identified eight SFKs in addition to Lyn: Src, Lck, Hck, Fyn, Blk, Fgr, Yes, and Yrk (28). Lyn is most closely related to Hck (29) and is highly conserved across species (30–32). Src, Fyn, and Yes are ubiquitously expressed, whereas Hck, Fgr, Blk, and Lck are expressed only in hematopoietic lineages (33). Lyn is expressed broadly, detectable in hematopoietic stem cells (HSC), B cells, myeloid cells, NK cells, endothelial cells, epithelial cells, and neurons (34, 35). Lyn, however, is not expressed in conventional T cells, being downregulated at the thymocyte double negative (DN) stage of T cell development (**Figure 1**).

**Figure 2:**
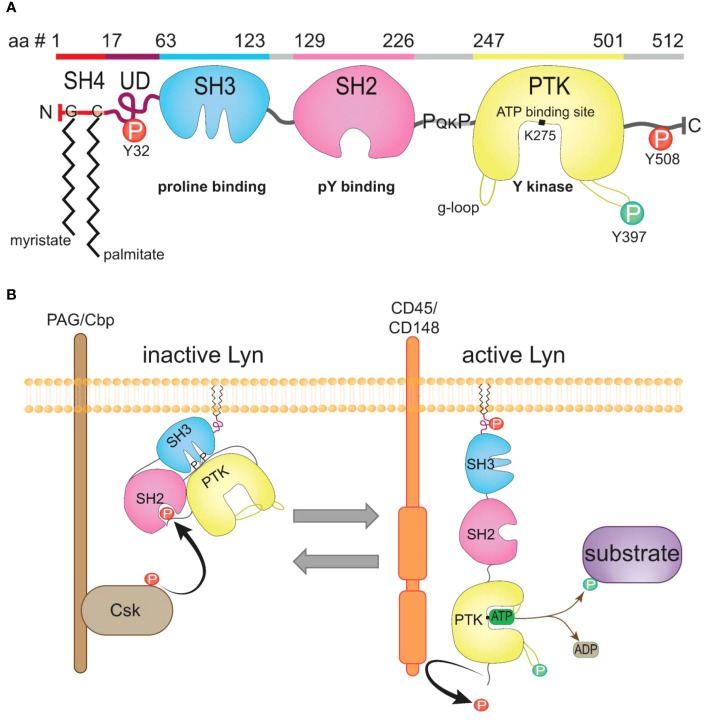
The structure of Lyn kinase and regulation of its activity. **(A)** The canonical LynA protein sequence; amino acid (aa) residues 1-512, with the domains highlighted and the corresponding schematic of structure depicted underneath (sequence from UniProt [31)]. Inhibitory pY residues (Y32, Y508, red), activating pY residue (Y397, green), the ATP-binding site (K275), myristate addition to residue G2 and palmitate to C3, and the SH3 proline-binding motif (P_QK_P) within the SH2-kinase linker sequence indicated. **(B)** Schematic of the active and inactive conformations of Lyn. Lyn is phosphorylated by Csk on Y508, placing Lyn in a closed inactive conformation. Lyn is activated by phosphatases such as CD45, which dephosphorylate Y508 on Lyn, removing the binding site for the SH2 domain, causing Lyn to unwind to an open and active conformation with ready access to its substrates. Lyn auto-phosphorylates Y397 to displace the activation loop from the active site, with the g-loop stabilizing ATP binding to K275 to enable phosphotransfer from ATP to substrate. Figure inspired by (37, 38).

Lyn, as with other SFKs, is a modular protein, containing four Src homology (SH1-4) domains and a unique domain (UD), comprehensively reviewed by others (28, 36) (**Figure 2A**). Two Lyn isoforms occur due to alternative splicing of Lyn pre-mRNA; a p56Lyn isoform (LynA) contains a 21-amino acid UD insert while the p53Lyn isoform (LynB) lacks this amino acid stretch (32, 39). Lyn is predominantly embedded in the intracellular side of the plasma membrane through the co-translational addition of saturated fatty acids to the N-terminus of Lyn (40, 41) (**Figures 2A, B**). The C-terminal end of Lyn comprises the protein tyrosine kinase (PTK) domain and a short tail containing the regulatory tyrosine residue (Y508) that determines Lyn’s activation status; when phosphorylated by the kinase Csk, pY508 places Lyn in an inactive closed conformation that blocks substrate access to the PTK (**Figure 2B**). Lyn is activated by dephosphorylating Y508 by phosphatases such as CD45 and CD148 (42), allowing Lyn to adopt its open conformation to allow for substrate phosphorylation to occur [reviewed by (37)] (**Figure 2B**).

Lyn plays a dual role in immune cell signaling by phosphorylating both activating and inhibitory immunoreceptors. In addition to Lyn’s known role in plasma membrane receptor phosphorylation, Lyn also phosphorylates substrates whilst in the Golgi apparatus during oxidative stress (43). A fraction of endogenous Lyn is also found in the nucleus (44, 45) where it can be activated by DNA damage, causing Lyn to phosphorylate inhibitory and activating nuclear targets (45–47). For activating receptors, the tyrosine motifs phosphorylated by Lyn and other SFKs are termed immunoreceptor tyrosine-based activation motifs (ITAMs) and have the conserved sequence YxxL_6-10_YxxL (48). Phosphorylated ITAMs act as binding sites for the recruitment and activation of downstream kinases such as Syk in B cells and myeloid cells and ZAP-70 in T cells (49, 50). Docking of Syk triggers its activation through autophosphorylation (51) and by direct phosphorylation via SFKs including Lyn (49). Active Syk initiates the activation of growth/inflammatory signaling pathways, including those regulated by mitogen-activated protein kinase (MAPK), nuclear factor kappa-light-chain-enhancer of activated B cells (NF-κB), and phosphatidylinositol 3-kinase/protein kinase B (PI3K/Akt).

Although other SFKs can phosphorylate inhibitory receptors (52, 53), Lyn plays a key role in inhibitory signaling whereupon it phosphorylates tyrosine residues in the conserved sequence I/V/LxYxxL/V, known as immunoreceptor tyrosine-based inhibitory motifs (ITIMs) (54). In contrast to ITAMs, ITIM phosphorylation recruits phosphatases to the plasma membrane and triggers their activation, which allows them to dephosphorylate and thereby turn off targets in the pathways activated by ITAM-recruited kinases (38, 54). ITIM-recruited phosphatases contain the SH2 phospho-tyrosine binding domain and include the lipid phosphatases SHIP-1/SHIP-2 and the protein tyrosine phosphatases SHP-1/SHP-2. As with Syk, Lyn also directly phosphorylates SHIP and SHP to cause their activation (55, 56), therefore phosphorylation of ITIM-containing inhibitory receptors typically occurs upon co-ligation with an activating receptor (57). Of note, SHP-1 and SHIP-1 can also be recruited directly to activating receptors to inhibit signaling; SHP-1 has been shown to bind IFNAR1, causing the dephosphorylation of associated Janus kinases and signal transducer and activator of transcription factors (STATs) (58, 59). Similarly, SHIP-1 is recruited to the M-CSF receptor (CSF-R1) to inhibit Akt phosphorylation (60, 61).

Interestingly, Lyn has also been shown to propagate inhibitory signaling from activating receptors, such as the FcαRI, FcγRIIa, and FcγRIIIa, with these signals termed inhibitory ITAM signals (ITAMi) (62–64). This occurs in the absence of receptor aggregation, or with low affinity binding to ligand (62, 64).

### 2.1 Activating signaling

**Figure 3:**
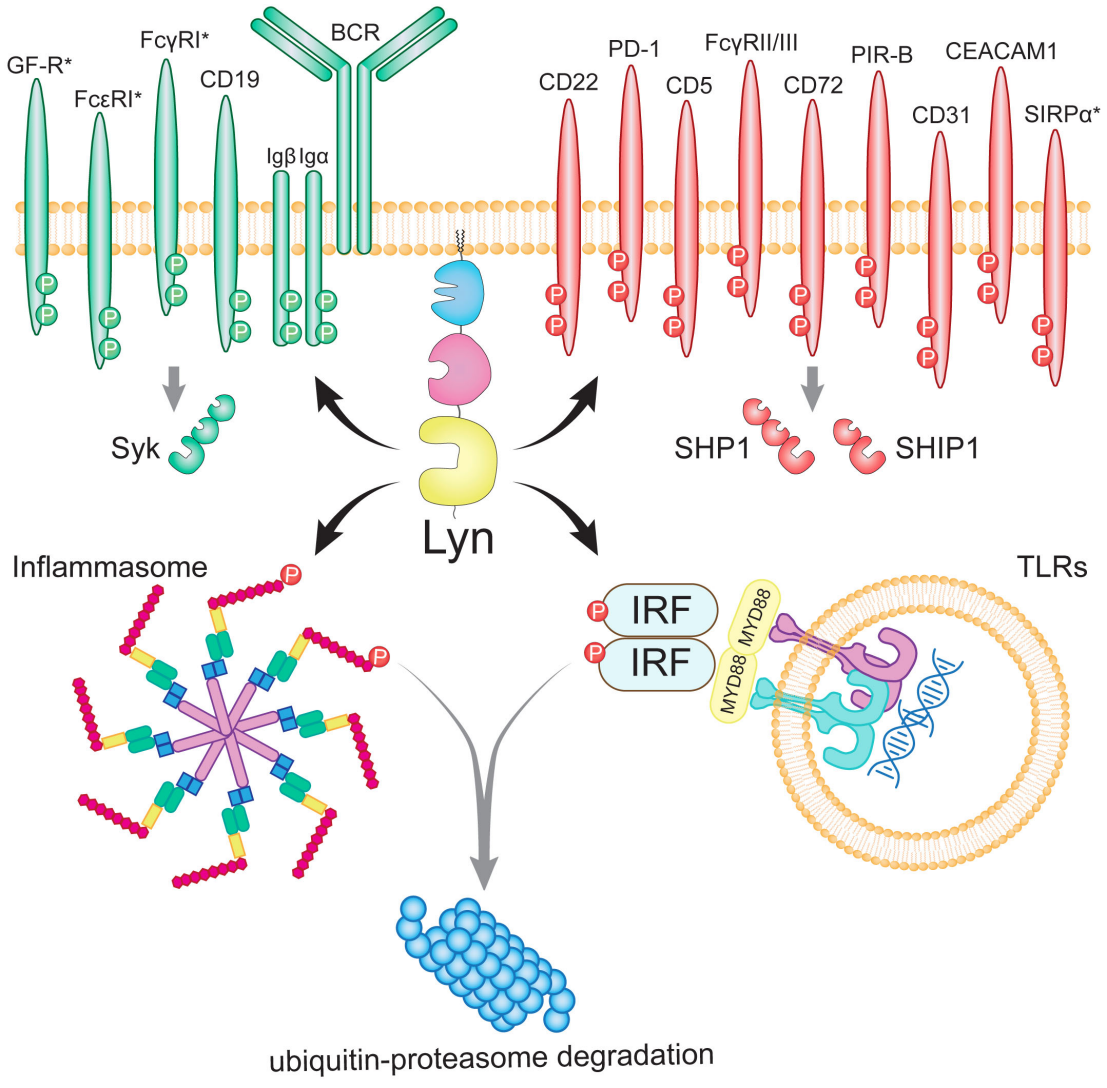
The network of Lyn’s phosphorylation targets in B lymphocytes and myeloid cells. Lyn drives activation by phosphorylating the ITAMs of activating receptors and Syk (indicated by green). Paradoxically, Lyn also promotes inhibition by phosphorylating the ITIMs of inhibitory receptors and SHP-1 and SHIP-1 (indicated by red). Lyn further promotes inhibition by phosphorylating the NLRP3 inflammasome (71) and the IRF family of transcription factors downstream of TLRs to target them for ubiquitin-proteasome degradation (72). Lyn has additionally been suggested to inhibit IRF5 through a protein-binding interaction (73). Myeloid-specific receptors (SIRPα, FcγRI, FcϵR and growth factor receptors; GF-R) are denoted with asterisks. Figure inspired by (37, 38).

Lyn colocalizes with, and phosphorylates ITAMs of numerous activating receptors including Igα/BCR (65), CD19 (66), high affinity FcϵRl complex (67), high affinity FcγRI (CD64) (68, 69), and Fc Receptor Like 5 (FCRL5) (70) (**Figure 3**). Lyn also colocalizes with and propagates signaling from activating receptors that do not contain an ITAM, including the Epo receptor (74, 75), the G-CSF receptor (76–78), the β-chain of IL-3/5/GM-CSF receptors (79), CD14 (80) [the LPS co-receptor with TLR4 and MD-2 (81)], CD36 (a scavenger receptor implicated in both atherosclerosis and amyloid plaque accumulation) (82), c-Kit (83), IL-2R (84), CD40 (85), and FLT3 (86, 87).

The activating role of Lyn is exemplified by studies using mice expressing a constitutively active Lyn mutant, termed Lyn^up/up^ mice. The Lyn^up^ mutant was generated through a gain-of-function point mutation (p.Y508F), which removes the regulatory tyrosine residue in Lyn, resulting in Lyn being unable to ‘switch off’ and thereby having constitutive kinase activity (88). Lyn^up/up^ B cells exhibit chronic activation due to continuous ITAM phosphorylation by Lyn, seen in basal phosphorylation of Syk and PLCγ2, with B cells displaying a hyperactive phenotype and increased calcium influx upon stimulation (88, 89). Due to immune cell hyperactivation, Lyn^up/up^ mice develop lupus-like disease including glomerulonephritis (90), as well as severe inflammatory lung disease (91).

Highlighting the duplicitous role of Lyn in signaling, Lyn^up/up^ immune cells also show constitutive inhibitory signaling due to ITIM phosphorylation by Lyn, including phosphorylation of the inhibitory receptors CD22, FcγRIIB, SIRPα, PIR-B, as well as SHIP-1 and SHP-1 (90). However, the severe autoimmunity and autoinflammation exhibited by Lyn^up/up^ mice demonstrates that chronic Lyn-driven activating signaling overpowers Lyn’s inhibitory-driven role. Yet, how Lyn gain-of-function mutations result in immune hyperactivation despite enhanced inhibitory signaling remains unclear, with further comparative studies of Lyn loss-of-function and Lyn gain-of-function mice warranted to gain additional mechanistic insight.

*De novo* gain-of-function mutations in LYN have also been reported in four patients, including two missense mutations [p.Y508H (92) and p.Y508F (93)] and two nonsense mutations (p.Y508* and p.Y507*) (93) that lead to a truncated LYN protein, lacking the Y508 regulatory residue. Echoing the phenotype of Lyn^up/up^ mice, gain-of-function mutations in patients caused severe systemic inflammation, with disease onset occurring from birth (92, 93). Features of disease included fever, cutaneous neutrophilic vasculitis/atopic dermatitis, arthritis/arthralgia, colitis, and liver damage (92, 93). Patients exhibited elevated circulating proinflammatory cytokines and C-reactive protein (92, 93), as well as the presence of autoantibodies (93). Functional studies revealed enhanced activation of B cells, neutrophils, macrophages, and endothelial cells (93). Patients were treated with anti-IL-1β, anti-TNFα, as well as the SFK kinase inhibitor dasatinib, which improved systemic inflammation (92, 93).

Enhanced Lyn kinase activity has also been observed in a truncated N-terminal mutant of Lyn (LynΔN) that forms during apoptosis via direct cleavage by executioner caspases, relocating Lyn to the cytosol and nucleus (94–96). LynΔN is proinflammatory, with Lyn^ΔN/ΔN^ mice developing a severe psoriasis-like disease that is dependent on TNFα and inflammatory caspase induction of IL-1β and IL-18 activation (95, 97). Biopsies of human psoriatic skin lesions similarly show increased expression of caspases and LynΔN, implicating cleaved Lyn in human psoriasis (97).

Of the two Lyn isoforms, LynA has been implicated as having a greater role in activation. The UD insert in LynA contains a tyrosine residue (Y32) (**Figure 2A**) that is phosphorylated by active Lyn and Hck in trans (98), as well as by the epidermal growth factor receptor in epithelial cells (99). Phosphorylation of Y32 targets LynA for polyubiquitination by c-Cbl, causing rapid LynA degradation following activation (98), which is consistent with reports of reduced LynA levels after B cell activation (39). The rapid degradation of LynA has been shown to prevent Lyn-driven hyperactivating signaling in macrophages (100). This can be fine-tuned by increasing Lyn protein levels by inducing *Lyn* transcription with IFNγ or GM-CSF priming, which compensates for the reduction in LynA, leading to enhanced Lyn-driven macrophage activation to subsequent stimulation (100). The divergent function of the isoforms has been further explored *in vivo* by the Freedman group through the generation of mice expressing a single isoform; either LynA or LynB (101). This work demonstrated that the co-expression of both isoforms is required to prevent lupus-like disease; however, LynB-deficient mice developed more severe autoimmune pathology compared to LynA-deficient mice, with greater incidence of ANA detection and severe glomerulonephritis (101). This suggests that LynB may play a greater role in inhibitory signaling, and LynA may be more important for activating signaling (101). This is further supported by reports showing that the LynA isoform is overexpressed in cancerous cells, implying a dominant role for LynA in activation (99, 102).

### 2.2 Inhibitory signaling

Generally, the role of Lyn in activating signaling can be compensated for by other SFKs, whereas its function in inhibitory receptor signaling in B lymphocytes and myeloid cells is non-redundant, leading to a loss of immune cell inhibition and resultant lupus-like disease in Lyn-deficient mice (Lyn^-/-^) (103, 104). Lyn^-/-^ mice are a well-studied model of lupus, exhibiting an age-dependent increase in pathogenic autoreactive antibodies including anti-dsDNA and anti-Sm IgG2b/c (105), IgA (106), and to a lesser extent IgE (107, 108). This promotes pathogenic immune complex deposition in the glomeruli, leading to recruitment of complement factor 3 deposition which drives inflammatory immune cell infiltration, culminating in glomerulonephritis. At the cellular level, Lyn deficiency results in hyperactive B cells to BCR crosslinking (109) and myeloid cells with enhanced TLR signaling (73, 110–112), integrin signaling (113, 114) and growth factor sensitivity (115).

B cells are necessary for autoimmune pathology in Lyn^-/-^ mice by generating class-switched autoantibodies, as the disease is abrogated by a block in B cell development (Lyn^-/-^μMT^-/-^) (106, 116). Autoimmune pathology is also T cell-dependent, as Lyn^-/-^ mice with an impairment in T cell development (Lyn^-/-^TCRβ^-/-^TCRδ^-/-^) or mice lacking the SAP signaling adaptor protein (Lyn^-/-^SAP^-/-^), which is critical for T cell-B cell interactions, both show attenuated production of anti-nuclear IgG autoantibodies (117). Given that T cells do not express Lyn, they are believed to be intrinsically unaffected in Lyn^-/-^ mice, with their hyperactivation driven by other immune compartments and the inflammatory milieu. Supporting this, lethally irradiated Lyn^+/+^ mice reconstituted with mixed Lyn^-/-^Rag^-/-^ and Lyn^+/+^ bone marrow exhibit T cell activation and autoimmune pathology, and adoptive transfer of Lyn^+/+^ T cells into aged Lyn^-/-^ mice results in their effector memory differentiation and IFNγ production on par with host Lyn^-/-^ T cells (111).

The autoimmune pathology seen in the global Lyn-deficient mice can be recapitulated by conditional deficiency of Lyn in B cells (Cd79a-cre x Lyn^flox/flox^) (105), myeloid cells (Lyn^−/−^Rag^−/−^- Lyn^+/+^ chimera) (111), as well as CD11c+ cells (CD11c-cre x Lyn^flox/flox^), which encompasses dendritic cells, age-associated B cells, type I innate lymphoid cells, macrophages, and patrolling monocytes, with the disease in fact being more severe in CD11c-cre x Lyn^flox/flox^ mice (112). Reflecting the inflammatory nature of the disease, pathology is dependent on inflammatory cytokines and is attenuated by IL-6 deficiency (106), IFNγ deficiency (111), IFNAR-1 deficiency (118), or treatment with neutralizing anti-BAFF antibody (111). Disease has repeatedly been shown to depend on toll-like receptor (TLR) signaling, as knockout of the MyD88 TLR adaptor protein abrogates disease (105, 112, 117, 119), as does disruption of the pattern recognition receptor adaptor protein CARD9 (120).

*Lyn* is also a haploinsufficient gene, with a loss-of-function in one allele (Lyn^+/-^ mice) sufficient to cause autoimmune disease with age (121). Disease in Lyn^+/-^ mice can also be accelerated to levels comparable to Lyn^-/-^ mice by concordant loss-of-function of one allele of *SHP-1* (Lyn^+/−^Me^v+/−^) or one allele of *SHIP-1* (Lyn^+/−^SHIP-1^+/−^) (121), reflecting the polygenic additive model of SLE heritability.

A growing list of inhibitory targets of Lyn, including ITIM-containing receptors, have been identified and have themselves been implicated in SLE and autoimmune pathology (**Figure 3**). The collective loss of inhibitory signaling emanating from these receptors upon Lyn deficiency provides a mechanistic framework for the Lyn^-/-^ autoimmune phenotype, which is largely driven by autoreactive B cells and hyperactive myeloid cells.

#### 2.2.1 Inhibitory Lyn targets expressed by B cells

*CD22* is expressed by B cells and is a member of the sialic-acid-binding immunoglobulin-type lectin (Siglec) family, which bind sialic acid on glycoproteins and lipids (122). Sialic acids are ubiquitous on vertebrate cells, and therefore act as a marker of self (123). Lyn is required for the ITIM phosphorylation of CD22 in order to recruit SHP-1 (124, 125) and to a lesser extent SHIP-1 (126). Co-ligation of the BCR and CD22 sends a ‘binding to self’ signal and therefore raises the activation threshold (127). Siglec-G is another B cell-specific Siglec that is believed to be similarly phosphorylated by Lyn (128, 129). CD22 deficiency alone causes an age-related expansion of autoreactive B cells without pathology (130), whilst double deficiency of both CD22 and Siglec-G results in glomerulonephritis (131). Sequence variation screening in SLE identified several substitutions in CD22 that were weakly associated with SLE (132), although this is not supported by genome-wide association studies (GWAS) (127). However, higher CD22 expression on circulating B cells has been associated with remission and low disease activity in SLE patients (133, 134).

*Programmed cell death receptor-1 (PD-1)* is an ITIM-bearing receptor that is expressed by B cells, activated T cells and T follicular helper cells (135). PD-1 binds to PD-L1 and PD-L2 on antigen-presenting cells (APCs), endothelial cells and epithelial cells (135) and upon co-ligation with the BCR, is phosphorylated by Lyn to induce the activation of SHP-2 (136). PD-1 inhibits B cell activation to CpG (TLR9 agonist) and anti-IgM stimulation (137), and T cells to TCR binding as well as modulating autoreactive T cell migration (135). PD-1-deficient mice develop SLE-like glomerulonephritis (138) with a number of studies identifying aberrant PD-1 and PD-L1/2 expression in SLE patients (139).

*CD5* is a member of the Scavenger Receptor Cysteine-Rich family and is an ITIM-containing receptor constitutively expressed by mature T cells (140), anergic autoreactive B cells (141), a subset of IL-10-producing regulatory B cells (142, 143), and B1a cells. CD5 has been demonstrated to bind itself *in cis* and *trans* (144), as well as to the common inhibitory receptor, CD72 (145). B1 cells are an innate-like B cell population that have a degree of self-reactivity, producing the majority of baseline circulating IgM and IgA antibodies and, although they have protective functions, are implicated in autoimmune pathology (146, 147). CD5 is phosphorylated by Lyn in B1 cells, leading to the recruitment and activation of SHP-1 to negatively regulate BCR signaling and promote B1 cell apoptosis upon BCR cross-linking (148, 149). B1a cell numbers are expanded in Lyn^up/up^ mice (90) and Lyn^-/-^ mice (105), likely reflecting perturbed CD5 signaling in Lyn-mutant mice.

#### 2.2.2 Inhibitory Lyn targets expressed by both B cells and myeloid cells

*FcγRIIb* is the only inhibitory member of the FcγR family, which are receptors that bind the constant region of IgG (150). FcγRIIb contains a single ITIM motif that is phosphorylated by Lyn and recruits SHIP-1 (109, 151). B cells, plasma cells, plasmacytoid dendritic cells (pDCs) (152), neurons (153) and some memory CD8+ T cells (154, 155) express the FcγRIIb only, whereas myeloid cells also express the activating FcγRs (FcγRI, FcγRIII, FcγRIV) in addition to FcγRIIb (150, 156). FcγRIIb inhibits B cells upon co-ligation with the BCR, and prevents the hyperactivation of myeloid cells to immune complex binding (156). In pDCs, FcγRIIb prevents viral-immune complex uptake from triggering IFNα production (157), whilst in conventional dendritic cells (cDCs), FcγRIIb prevents spontaneous maturation in response to sterile immune complexes (158–160). Given its far reaching inhibitory functions, it is perhaps unsurprising that FcγRIIb-deficient mice develop severe lupus-like nephritis, with 60% mortality by 9-months-of-age (161). The overexpression of FcγRIIb has also been shown to ameliorate disease in other lupus models (162, 163), with *FCGR2B* being a strong susceptibility gene in SLE (164–166). Given the broad functions of FcγRIIb, loss of signaling emanating from this receptor in Lyn-deficient mice likely has wide-ranging effects contributing to the Lyn^-/-^ phenotype.

*Paired immunoglobulin Receptor-B (PIR-B)* is an ITIM-containing receptor expressed in B cells, myeloid cells, and neurons, that binds to MHC-I *in cis* and *trans* (167). Lyn constitutively phosphorylates PIR-B to recruit and activate SHP-1 (88, 168) but additionally phosphorylates PIR-B in response to TLR9 stimulation with CpG-B (169). PIR-B has broad-ranging inhibitory functions, including negatively regulating TLR-induced B1 cell proliferation, B2 cell proliferation to BCR cross-linking (170), and myeloid cell integrin (171) and chemokine signaling (52), as well as proinflammatory macrophage polarization (172). PIR-B-deficient mice do not develop spontaneous autoimmune disease (169, 170); however, PIR-B deficiency on the C57BL/6-Fas^lpr^ background (an autoimmune susceptibility mutant), results in glomerulonephritis associated with enhanced production of rheumatoid factor autoantibodies by B1 cells (169). In DCs, PIR-B deficiency results in an immature phenotype (170, 173) and Th2-skewed responses (170); however, PIR-B-deficiency also enhances DC cross-presentation to cytotoxic T cells (173), and pDC production of IFNα to TLR9 stimulation with CpG-A (174). Therefore, in addition to promoting the hyperactivation of B lymphocytes and myeloid cells, diminished PIR-B signaling in Lyn^-/-^ mice will also impact the DC compartment.

*CD72* is a C-type lectin-like domain receptor expressed by B cells, pDCs, cDC2s, macrophages, and NK cells (35, 57, 175) and binds the lupus self-antigen ribonucleic protein Sm (176). The ITIM of CD72 is phosphorylated by Lyn and recruits SHP-1 to inhibit B cell activation upon co-ligation with the BCR (176, 177). CD72-deficient mice develop a spontaneous lupus-like disease with age (178). Further, a CD72 mutant with impaired ability to bind Sm/RNP exacerbates lupus-like disease on a susceptible background (176, 179). CD72 SNPs have also been associated with SLE (180), and B cells from SLE patients show reduced CD72 expression which correlates with disease activity (181, 182). CD72 also binds free CD100 (Semaphorin-4D), which is expressed ubiquitously, and is cleaved from the cell surface during activation, with soluble CD100 inhibiting myeloid cell activation through CD72 binding (175).

*NLRP3* inflammasomes are multi-protein complexes that assemble in the cytoplasm in response to a wide range of stimuli and induce the maturation of caspase-1, leading to activation of the proinflammatory cytokines IL-18 and IL-1β in B cells, myeloid cells, and epithelial cells (183, 184). Lyn has been shown to phosphorylate NLRP3 causing its ubiquitination and degradation (**Figure 3**), with Lyn-deficient macrophages hyperproducing IL-1β (71). Further, Lyn activation downstream of BCR cross-linking also attenuates inflammasome formation (185). Impaired inhibition of NLRP3 in Lyn^-/-^ mice is therefore likely a key contributor to the autoimmune pathology that develops.

#### 2.2.3 Inhibitory Lyn targets expressed by myeloid cells

*SIRPα* is a receptor of the signal regulatory protein (SIRP) family, being the only ITIM-bearing member (186), and is expressed on monocytes, macrophages, microglia, neutrophils, NK cells, neurons, pDCs and cDC2s (35, 186, 187). SIRPα is phosphorylated by Lyn to recruit SHP-1 and SHP-2 (88, 188, 189). Upon ligation with CD47, which is an integrin-associated protein expressed by all cells that acts as a marker of ‘self’, SIRPα sends a ‘don’t-eat-me’ signal to phagocytes (186), particularly in the context of inflammation (190, 191). SIRPα-deficient mice do not exhibit an overt phenotype at the steady-state (190). However, SIRPα suppresses severe systemic inflammation upon challenge with TLR-agonists and cytokines by attenuating macrophage activation (191). Whilst SIRPα has been shown to suppress inflammation, the role of SIRPα in autoimmunity is somewhat mixed, as SIRPα promotes DC priming of autoreactive T cells in collagen-induced arthritis (192), experimental autoimmune encephalomyelitis (193), and nonobese diabetic mice (194); however, SIRPα agonism conversely suppresses monocyte and neutrophil infiltration in experimental arthritis and colitis (195).

#### 2.2.4 Myeloid-specific Siglec receptors

Given the role of Lyn in CD22 and Siglec-G ITIM phosphorylation, it is likely that Lyn is also implicated in the phosphorylation of other Siglec receptors. Siglec-F is an ITIM-containing receptor expressed by eosinophils, alveolar macrophages, microglia (196) as well as allergen-induced CD4+ T cells and lung stroma (197). Siglec-F is pro-apoptotic in eosinophils, with Siglec-F-deficient mice exhibiting eosinophilia upon allergen induction (197). Similarly, Lyn is also pro-apoptotic in eosinophils, with Lyn^-/-^ mice exhibiting peritoneal eosinophilia (108) and lung eosinophilia upon ovalbumin challenge (198), and the treatment of eosinophils with antisense oligos to Lyn inhibits apoptosis (199). Furthermore, studies have implicated Lyn in microglial Siglec-F signaling, as upregulation of Siglec-F on microglia during neurodegenerative disease models is associated with increased activation of Lyn (196). However in eosinophils, Lyn deficiency does not perturb apoptosis to Siglec-F agonism compared to WT eosinophils (200). Therefore, whilst Lyn may be implicated in Siglec-F signaling, the details of Lyn’s involvement have not been elucidated.

Siglec-H is expressed by pDCs, microglia, DC progenitors, as well as intracellularly by marginal zone macrophages (201, 202) and triggers endocytosis upon ligation (201, 203). Siglec-H has been shown to inhibit pDC activation and IFNα production (204–206). Interestingly, Siglec-H deficiency outside of pDCs is sufficient to cause IFNα hyperproduction, as Siglec-H-deficient mice depleted of pDCs still hyperproduce IFNα during viral challenge (202). Siglec-H deficiency in mice results in mild autoimmune disease, and early infection by murine cytomegalovirus causes the development of severe lupus-like glomerulonephritis, which is dependent on IFNAR1 (206).

Unlike other Siglec receptors, Siglec-H does not contain an ITIM in its cytoplasmic tail (123). Instead, Siglec-H recruits DAP12, an ITAM-containing adaptor protein (203) that can be phosphorylated by SFKs (207). Given that Lyn^-/-^ pDCs hyperproduce IFN-I in response to CpG (73, 112), it is possible that Lyn is involved in Siglec-H signaling, and that impaired Siglec-H signaling contributes to hyperproduction of IFN-I in Lyn^-/-^ mice. However, whilst the SFK Hck has been ruled out as being involved in Siglec-H signaling (208), involvement of Lyn in Siglec-H signaling has not yet been assessed.

#### 2.2.5 Inhibitory Lyn targets expressed ubiquitously

*Platelet endothelial cell adhesion molecule-1 (PECAM-1/CD31)* is an Ig-like superfamily receptor which is highly expressed on endothelial cells towards the tight junctions, and to a lesser degree on most leukocytes and platelets (35, 209). PECAM-1 binds itself *in trans*, in addition to numerous other identified ligands, and is an ITIM-bearing receptor (209, 210). Lyn is required for the phosphorylation of PECAM-1 to recruit SHP-1/SHP-2 and phospholipase Cγ1 (210, 211). PECAM-1 is involved in leukocyte recruitment through homophilic binding between endothelial cells and leukocytes, initiating leukocyte diapedesis, which is largely independent of PECAM-1’s cytoplasmic signaling (210). PECAM-1 has also been shown to inhibit FcϵR signaling in mast cells (212), as well as FcγR (213) and TLR4 signaling (214) in human monocytes and macrophages. However, it is less clear whether the inhibitory function of PECAM-1 is recapitulated in mouse macrophages (215). PECAM-1 deficiency also causes hyperactivation of B cells to BCR cross-linking, with PECAM-1-deficient mice exhibiting reduced B2 cell numbers in the periphery and increased peritoneal B1 cells, as well autoantibody production, culminating in glomerulonephritis (216) – all features shared by Lyn^-/-^ mice. Interestingly, Lyn has been implicated in strengthening epithelial barrier integrity (217), which may in part be through propagating PECAM-1 signaling, as PECAM-1 is required for maintaining epithelial barrier integrity (210, 218).

*Carcinoembryonic antigen-related cell adhesion molecule 1 (CEACAM1)* is the only ITIM-bearing member of the CEACAM family, and while it is constitutively expressed on epithelial cells, it is upregulated by activated leukocytes and endothelial cells (219). CEACAM-1 primarily binds itself *in cis* and *trans*, and acts as an inhibitory co-receptor, bringing phosphatases SHP-1/SHP-2 to the immune synapse to inhibit activation, and is important in preventing neutrophil, monocyte, B, T, and NK cell hyperactivation (219, 220). Lyn has been shown to phosphorylate the ITIM in CEACAM1 in myeloid cells (221, 222) and is important in myelopoiesis, with CEACAM1^-/-^ mice having pronounced neutrophilia due to hyperresponsiveness to G-CSF (223). CEACAM1 deficiency also causes a reduction in Ly6C+ monocyte numbers during infection (224). Both neutrophilia and a reduction of Ly6C+ monocytes are features of Lyn^-/-^ mice (225, 226), suggesting that perturbed CEACAM1 potentially contributes to the phenotype of Lyn-deficient mice.

#### 2.2.6 Inhibitory protein-binding targets of Lyn independent of kinase activity

In addition to Lyn’s tyrosine kinase function, there is also evidence that Lyn has a kinase-independent role through inhibitory protein-binding interactions.

Two Lyn mutants with reduced or no detectable kinase activity have been generated through ENU mutagenesis (227, 228). The p.T410K mutation destabilizes the activation loop, causing no detectable Lyn kinase activity, conceivably by preventing displacement of Lyn’s activation loop and thereby blocking substrate access to the active site (227). The p.E260G (228) mutation destabilizes the g-loop and the entire kinase domain, resulting in significantly reduced kinase activity (228, 229). Lyn^T410K/T410K^ and Lyn^E260G/E260G^ B cells exhibit hyper-responsiveness to BCR cross-linking on par with Lyn^-/-^ B cells (227, 228). However, Lyn^T410K/T410K^ mice were reported to have attenuated autoimmune pathology (227), while Lyn^E260G/E260G^ mice have a delayed onset of pathology compared to Lyn^-/-^ mice (228), indicating that Lyn has undefined inhibitory functions independent of its kinase activity in suppressing autoimmune pathology *in vivo*. However, the modifications in Lyn^T410K^ and Lyn^E260G^ are not true kinase-dead mutations as they occur outside of the catalytic site. These mutations have the potential to alter substrate-binding specificity (73) while retaining residual kinase functionality (229). Therefore, characterizing mice expressing an authentic kinase-dead mutation that affects the ATP-binding residue (p.K275X) is necessary to assess the true protein-binding functions of Lyn *in vivo*. The Lyn mutant p.K275X has been studied *in vitro* (44, 73, 98); however, mice expressing Lyn p.K275X have not yet been reported.

A mechanistic description of an inhibitory protein-binding interaction with Lyn and the interferon regulatory factor 5 (IRF5) transcription factor was provided by Ban et al. in 2016 (73). IRF5 induces the transcription of proinflammatory cytokines including IL-6, IL-12, and IFN-I and is strongly implicated in SLE pathology, being routinely identified in GWAS (230). The alleles of *IRF5* associated with SLE induce the hyperactivation of IRF5 and cause spontaneous lupus-like disease in mice (231). Lyn was shown to bind IRF5 upon TLR7/9 stimulation downstream of the Myddosome oligomeric signaling complex (73). Lyn binding inhibited IRF5 activation by blocking access to IRF5 by the kinase IKKβ and the ubiquitin ligase TARF6 (73). This study showed that the kinase function of Lyn was dispensable for IRF5 inhibition, providing a potential kinase-independent mechanism that may explain the reduced pathology seen Lyn^T410K/T410K^ and Lyn^E260G/E260G^ mice. In further support of this, knockout of IRF5 was sufficient to attenuate TLR-induced cytokine production from DCs *in vitro* (Lyn^-/-^IRF5^-/-^), and monoallelic loss of IRF5 was sufficient to abrogate glomerulonephritis *in vivo* (Lyn^-/-^IRF5^+/-^) (73, 118), likely through suppressing transcription of proinflammatory cytokines, IFN-I, and oxidative phosphorylation pathway genes (118). This work also implicated a key role for this inhibitory function in the DC compartment, although analysis of DCs *in vivo* from Lyn^-/-^ mice was not performed (73).

Later studies have, however, challenged the protein-binding inhibition of IRF5 by Lyn, and instead showed that Lyn inhibits the IRF family (IRF1, IRF5, IRF7, and IRF8) by phosphorylating a conserved tyrosine residue (p.Y118 in IRF5), which inhibits their activity by targeting IRFs for ubiquitination and proteasomal degradation (72) (**Figure 3**). Further conflicting findings have suggested that Lyn instead promotes IRF5 activation and nuclear translocation (232). Given the contradictory literature, further work is required to clarify the role of Lyn in relation to the IRF family, and whether this interaction is a protein-binding interaction or a kinase interaction.

## 3 Studies of Lyn in SLE patients

Relative to the expansive number of experimental animal studies focused on Lyn, the role of Lyn in SLE patient disease has been under-researched; however, Lyn has similarly been implicated in human SLE disease.

Multiple *LYN* single nucleotide polymorphisms (SNPs) have been associated with SLE through GWAS. The SNPs rs7829816 and rs2667978 were first identified as being protective in SLE development in women of European descent in a 2008 GWAS, with an overall odds ratio of 0.77 and 0.81 respectively, although an association was not seen across all data sets analyzed (233). A replication study that analyzed 90 *LYN* SNPs in 2009 similarly detected associations for rs7829816 and rs2667978 in a pooled analysis of women of European ancestry, as well as a number of other *LYN* SNPs, though at a greatly reduced level of significance (234). Instead, this later study identified a stronger association with another SNP, rs6983130, which was associated with SLE with hematological presentations and the presence of autoantibodies including anti-dsDNA and anti-Sm (234). This study did not find an association for *LYN* in African-American and Korean populations, although these cohorts had a lower power of detection (234). A large-scale GWAS and meta-analysis conducted in 2015 on individuals of European descent similarly identified an association with the *LYN* SNP rs2667978 and SLE, although a strict genome-wide level of significance was not met (235). Similarly, a 2018 Spanish GWAS and meta-analysis of European descent individuals found an association between another *LYN* SNP rs17812659 and SLE, although again short of genome-wide level of significance (236). Collectively, GWAS have provided evidence for a genetic role of *LYN* in SLE, although this is weaker compared to routinely identified genes. However, all identified *LYN* SNPs reside in non-coding regions of the *LYN* gene without functional annotations, making implications of *LYN* SNP-associations with SLE unclear.

Studies have also investigated perturbations in LYN expression in SLE patients. Reduced LYN protein levels have been found in resting and activated circulating B cells in the majority of SLE patients, which was stable over time and independent of disease activity (237, 238). This was associated with reduced *LYN* mRNA expression (237, 239) as well as increased ubiquitination of the LYN protein (238), suggesting multiple mechanisms for reduced LYN levels in SLE B cells [reviewed by (240)]. These findings indicate that reduced Lyn activity through under-expression of Lyn may contribute to SLE pathogenesis in a manner similar to the disease process of Lyn^+/-^ mice (121). In support of this, SNPs conferring elevated expression of CSK, the negative regulator of Lyn activity, have been identified in SLE patients and cause enhanced Lyn kinase inhibition and associated B cell hyperactivation (241). However, the aforementioned studies of LYN in SLE patients have focused on B lymphocytes, whilst myeloid cells were omitted from analyses (237–239). Therefore, it is unclear whether the status of LYN expression in SLE B lymphocytes is representative of LYN perturbations across the entire immune system in SLE; an important unanswered question given the critical role of Lyn in regulating activation of the myeloid compartment.

Recent RNA-sequencing and microarray studies in SLE patients have also contradicted earlier findings, instead reporting increased *LYN* mRNA expression in SLE patients. RNA-sequencing of antibody-secreting cells from SLE patients has revealed upregulated *LYN* expression compared to healthy controls (242). Similarly, a bulk RNA-sequencing analysis of SLE patient whole blood by Panousis et al. (2019) identified *LYN* as a differentially upregulated gene (243). Further, a longitudinal microarray analysis of pediatric SLE patient blood by Banchereau et al. (2016) linked increased *LYN* expression to disease activity in SLE patients, with expression quantitative trait loci analysis identifying seven SNPs in neighboring genes associated with the change in *LYN* expression (244). The baseline whole blood microarray analysis from SLE patients enrolled in the tabalumab (anti-BAFF) phase III trials also linked increased *LYN* expression to disease activity, IFN-I signature expression, and anti-dsDNA antibodies through weighted gene co-expression network analysis, identifying *LYN* in the ‘inflammatory response’ module (245). As previous work has shown that inflammatory cytokines induce the upregulation of *Lyn* in mouse macrophages to cause activation priming (100, 246), it is conceivable that SLE patients may exhibit augmented *LYN* expression, given the chronic inflammatory insult characteristic of SLE. In humans, *LYN* expression has also been found to be induced in autoimmune patient B cells by the use of TNFα blockers, resulting in enhanced Lyn activity and B cell hyperactivity, which was postulated to underlie the autoimmune syndrome that develops in a subset of TNFα-antagonist treated patients (247). However, SLE transcriptomic studies have not investigated LYN at the protein level and, therefore, LYN overexpression in SLE patients is yet to be substantiated.

## 4 Concluding remarks

Extensive studies in mice have illustrated the central role of Lyn in maintaining immune cell signaling balance by dualistically propagating both activating and inhibitory pathways. Beyond mouse models, there is a need to expand our understanding of LYN’s role in human SLE disease. Given Lyn can be pathogenic through both overexpression and under-expression, it is of benefit to further study the function of LYN in SLE disease. It is possible that perturbations in LYN may be cell type-specific and affect subsets of SLE patients. Potential therefore exists in repurposing drugs such as the Lyn inhibitor dasatinib (93) or the Lyn activator tolimidone (248) in a precision medicine approach to SLE treatment where LYN dysregulation is identified. Hence, additional studies are needed to consolidate differences in LYN expression in SLE patients as well as to discern the functional consequences of *LYN* SNPs and the veracity of Lyn as a biomarker for a precision medicine approach to SLE treatment.

## Author contributions

EL’E-S: Writing – original draft, Visualization, Investigation, Formal analysis, Conceptualization. TG: Writing – review & editing, Supervision. MW: Writing – review & editing, Supervision. MH: Writing – review & editing, Supervision, Resources, Project administration, Funding acquisition.
